# Case of Death Following the Excessive Use of Ardent Spirits; in Which Appearances Simulating Irritant Poisoning Were Found on Dissection

**Published:** 1845-01

**Authors:** John Inglis Nicol

**Affiliations:** Inverness


*Case of Death following the Excessive Use of Ardent Spirits; in in which Appearances simulating Irritant Poisoning were found on Dissection.* By John Inglis Nicol, M. D., Inverness.—J. F. a young man about 36 years of age, had indulged in drinking a considerable quantity of whiskey in company with some friends. On his return home, two of his companions accompanied him, as he seemed much intoxicated. They had walked but a short way up an acclivity, when he fell, uttered a few words, and immediately became quite insensible. His companions, supposing him merely to be very drunk, carried him home, and after very considerable difficulty they succeeded in placing him in his bed. They could not say whether he was drunk at the time; they only thought him to be drunk. He was found dead next morning.

Seven hours after he had been discovered in this state, a post mortem was instituted—a few scratches on left cheek—back and depending parts of the body very livid; and under the angles of the jaws and along the sides of the neck, the colour amounted to a deep purple.

Thoracic viscera healthy. In the abdomen the distention of the larger gastric and gastro-epiploic veins attracted immediate notice—liver congested. On opening stomach, there was an evident odour of ardent spirits. The œsophagus, stomach, and a large portion of the intestines being opened to view, the whole of the mucous membrane, from about half-way up the œsophagus, along the whole extent of the stomach and for about 18 inches along the intestines, was found highly injected. The vascularity of the corrugated part of the stomach was of a deep crimson color—œsophagus and intestines presented a like appearance. Whilst the head was being examined, a large quantity of liquid blood flowed from the divided vessels; apopletic congestion of the vessels of the investing membranes was very evident. The opinion given was that death was caused by apoplexy. After a careful examination, not a trace of irritant or corrosive poison could be detected. Dr. Yellowly has given a paper published in the fourth volume of the Transactions of the London Medical and Chirurgical Society, from which it would appear that such cases as the present are of more frequent occurrence than is generally supposed.—*London and Ed. Monthly Journal.*

